# National Estimates of Increase in US Mechanical Ventilator Supply During the COVID-19 Pandemic

**DOI:** 10.1001/jamanetworkopen.2022.24853

**Published:** 2022-08-02

**Authors:** Thomas C. Tsai, E. John Orav, Ashish K. Jha, Jose F. Figueroa

**Affiliations:** 1 Department of Surgery, Brigham and Women’s Hospital, Boston, Massachusetts; 2 Department of Health Policy and Management, Harvard T. H. Chan School of Public Health, Boston, Massachusetts; 3 Division of General Internal Medicine, Brigham and Women’s Hospital, Boston, Massachusetts; 4 Brown University School of Public Health, Providence, Rhode Island

## Abstract

This cross-sectional study assesses whether the increase in adult and pediatric and neonatal mechanical ventilators in US hospitals in response to the COVID-19 pandemic varied across hospital structural features, region, and safety-net status.

## Introduction

Despite the unprecedented demand for mechanical ventilators in the US during the COVID-19 pandemic, the last survey of US hospitals occurred in 2010 and estimated approximately 62 000 full-featured ventilators.^1^ To meet the demand for mechanical ventilation, the federal government invoked the Defense Production Act to partner with manufacturers to purchase as many as 200 000 ventilators of varying functionality across production phases for the US Strategic National Stockpile.^2^ Many hospitals also purchased additional full-featured ventilators during the pandemic. Therefore, we performed an updated assessment of current ventilators in use at US hospitals to aid with current and future pandemic preparedness and to determine whether any change was correlated with COVID-19 burden.

## Methods

In this cross-sectional study, we used new survey data from the 2020 American Hospital Association (AHA) Annual Survey to estimate the increase in mechanical ventilator supply. The ventilator questions were developed in response to the COVID-19 pandemic and were fielded for the first time in 2020. Data from the AHA were merged with US Census data and COVID-19 burden data from the Department of Health and Human Services for 2020 to correspond with the AHA survey year.^3^ We assessed whether the increase in adult and pediatric and neonatal mechanical ventilators in US hospitals varied across hospital structural features, region, and safety-net status using a multivariable regression model weighted by likelihood of survey response to create national-level estimates. We then created a map of current adult mechanical ventilators per 100 000 residents using the state-level population estimates from the 2020 US Census.^4^ A state-level analysis used Pearson statistics to assess the correlation between increase in ventilators per capita and COVID-19 intensive care unit (ICU) admissions per capita. Analyses were performed with SAS, version 9.4M7 (SAS Institute Inc). Two-sided *P* < .05 indicated statistical significance. This study was deemed exempt from review by the institutional review board of the Harvard T. H. Chan School of Public Health owing to the use of deidentified hospital-level data. We followed the STROBE reporting guideline.

## Results

In total, 2712 of 4609 US adult acute care hospitals responded to the survey (response rate, 58.8%). Responding hospitals were more likely to be large (367 [13.5%] vs 111 [5.9%]; *P* < .001), major teaching hospitals (207 [7.6%] vs 44 [2.3%]; *P* < .001), and not for profit (1887 [69.6%] vs 979 [51.6%]; *P* < .001). Of hospitals providing pediatric care, 1103 of 1397 responded (response rate, 79.0%). The multivariate adjusted relative increase in adult mechanical ventilators during the public health emergency in 2020 was 31.5% (95% CI, 22.4%-41.3%; *P* < .001) (Table). The statistically significant increase in adult mechanical ventilators during the COVID-19 pandemic did not vary across hospital characteristics (Table). The increase in pediatric and neonatal mechanical ventilators was 15.6% (95% CI, 1.6%-31.5%; *P* = .03).

**Table.** National Estimates of Increase in Adult Mechanical Ventilators in the US by Hospital Characteristics During the COVID-19 Pandemic, 2020^a^

Characteristic	Adult mechanical ventilator survey items					Pediatric and neonatal mechanical ventilator survey items				
	Responding hospitals, No. (%) (n = 2712)	No. of adult mechanical ventilators		Multivariate adjusted relative increase in ventilators, % (95% CI)	*P* value	Responding hospitals, No. (%) (n = 1103)	No. of pediatric and neonatal mechanical ventilators		Multivariate adjusted relative increase in ventilators, % (95% CI)	*P* value
		2019	2020				2019	2020		
All	NA	80 455	102 670	31.5 (22.4 to 41.3)	<.001	NA	16 303	22 641	15.6 (1.6 to 31.5)	.03
Size										
Small (<100 beds)	1313 (48.4)	9203	12 132	33.4 (20.7 to 47.4)	<.001	242 (21.9)	452	542	20.9 (−14.4 to 70.7)	.28
Medium (100-399 beds)	1032 (38.1)	35 278	46 319	31.1 (16.8 to 47.1)	<.001	545 (49.4)	6209	7013	13.2 (−4.4 to 34.1)	.15
Large (≥400 beds)	367 (13.5)	35 973	44 218	22.7 (−3.9 to 56.7)	.10	316 (28.6)	9642	11 109	17.9 (−7.4 to 50.2)	.18
Hospital region										
Northeast	362 (13.3)	15 485	21 530	35.0 (8.8 to 67.5)	<.01	173 (15.7)	2843	3654	29.6 (−7.5 to 81.8)	.13
Midwest	915 (33.7)	17 187	21 859	26.9 (10.1 to 46.3)	<.01	315 (28.5)	3731	4190	11.8 (−24.3 to 45.8)	.41
South	996 (36.7)	31 676	38 578	33.3 (18.6 to 49.8)	<.001	401 (36.3)	6128	6811	15.0 (−6.3 to 41.2)	.18
West	439 (16.2)	16 107	20 703	32.0 (14.4 to 52.4)	<.001	214 (19.4)	3601	4008	12.2 (−14.4 to 47.0)	.40
Profit status										
For profit	319 (11.8)	9310	11 857	27.3 (9.1 to 48.7)	<.01	130 (11.8)	1862	2219	15.4 (−14.9 to 56.5)	.36
Not for profit	1887 (69.6)	58 194	74 303	29.5 (17.5 to 42.7)	<.001	823 (74.6)	11 388	12 861	12.0 (−4.2 to 30.9)	.15
Public	502 (18.5)	12 821	16 263	41.3 (20.8 to 65.2)	<.001	150 (13.6)	3030	3559	36.2 (−3.9 to 93.1)	.08
Teaching status										
Major	207 (7.6)	20 587	26 307	26.6 (88.3 to 81.5)	.20	170 (15.4)	5988	6924	15.0 (−19.3 to 63.9)	.44
Minor	901 (33.2)	36 680	46 349	31.1 (14.9 to 49.7)	<.001	521 (47.2)	7878	8785	15.2 (−4.0 to 38.1)	.13
Nonteaching	1604 (59.1)	23 187	30 014	32.0 (20.8 to 44.2)	<.001	412 (37.3)	2437	2954	16.4 (−5.9 to 44.0)	.16
Critical access status										
No	1993 (73.5)	77 315	98 284	28.0 (17.6 to 39.3)	<.001	986 (89.4)	16 242	18 581	14.7 (0.5 to 30.9)	.04
Yes	719 (26.5)	3140	4386	41.2 (23.1 to 62.0	<.001	117 (10.6)	61	83	33.7 (−25 to 138.3)	.32
Location										
Rural	540 (19.9)	2753	3660	38.1 (18.7 to 60.8)	<.001	87 (7.9)	68	84	39.3 (−29.5 to 175)	.34
Urban	2172 (80.1)	77 702	99 010	29.7 (19.5 to 40.7)	<.001	1016 (92.1)	16 235	18 579	14.8 (0.7 to 30.9)	.04
Safety net										
No	2259 (83.3)	52 731	67 339	32.2 (21.7 to 43.5)	<.001	845 (76.6)	10 140	11 323	11.6 (−4.5 to 30.4)	.17
Yes	453 (16.7)	27 724	35 331	29.4 (11.4 to 50.3)	<.001	258 (23.4)	6163	7341	24.7 (−0.7 to 56.7)	.06

Abbreviation: NA, not applicable.

^a^ Comparisons of whether multivariate adjusted increase in adult mechanical ventilators varied across hospital characteristic were all statistically nonsignificant.

South Carolina, Alaska, Nevada, Idaho, and Mississippi had the lowest per capita supply of adult mechanical ventilators, whereas New York, Louisiana, Arkansas, North Dakota, and Washington, DC, had the highest per capita supply (Figure). The state-level increase in ventilators during the pandemic was not correlated with the state-level ICU burden (*r* = 0.13; *P* = .37).

**Figure.**
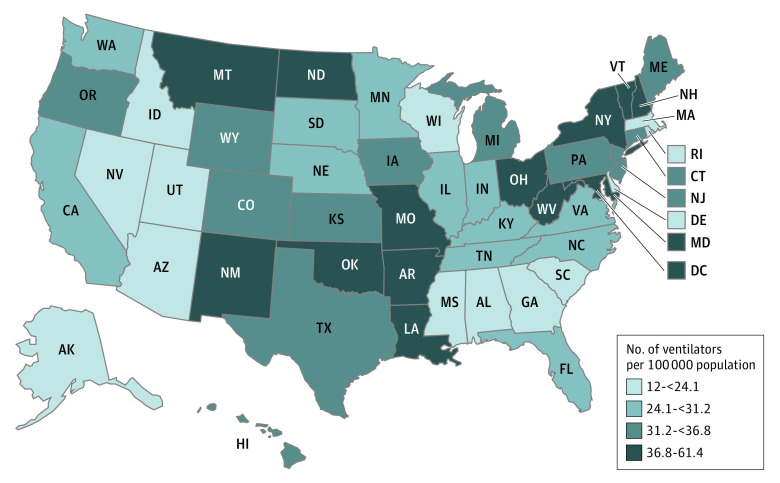
Total Adult Mechanical Ventilators in US Acute Care Hospitals per 100 000 Residents Data are from 2020.

## Discussion

This cross-sectional study found a substantial increase in adult and pediatric mechanical ventilators reported by acute care hospitals in 2020 compared with 2019. Study limitations include possible response bias. The AHA Annual Survey did not differentiate between ventilator functionality, changes in numbers of anesthesia ventilators, or transport, backup, or rental ventilators, nor did it account for temporary ventilator allocation coordinated through state public health departments or the Dynamic Ventilator Reserve.^5^ The increase in ventilators that were deployed in hospitals was not correlated with the state’s COVID-19 ICU burden, but this finding may not account for more dynamic shifts in ventilator stock across hospitals during waves of the pandemic. For both the current COVID-19 pandemic as well as future pandemic preparedness, these findings may help guide policy makers in deploying ventilators to states with the most urgent need of ventilators.

## References

[1] Rubinson L, Vaughn F, Nelson S, . Mechanical ventilators in US acute care hospitals. Disaster Med Public Health Prep. 2010;4(3):199-206. doi:10.1001/dmp.2010.18 21149215

[2] Branson R, Dichter JR, Feldman H, . The US Strategic National Stockpile ventilators in coronavirus disease 2019: a comparison of functionality and analysis regarding the emergency purchase of 200 000 devices. Chest. 2021;159(2):634-652. doi:10.1016/j.chest.2020.09.085 32971074 PMC7503115

[3] HealthData.gov. COVID-19 reported patient impact and hospital capacity by state. Updated June 27, 2022. Accessed January 19, 2022. https://healthdata.gov/Hospital/COVID-19-Reported-Patient-Impact-and-Hospital-Capa/g62h-syeh

[4] US Census Bureau. Historical population density data (1910-2020). Updated October 8, 2021. Accessed December 23, 2021. https://www.census.gov/data/tables/time-series/dec/density-data-text.html

[5] Keohane LM. Expanding ventilator capacity—the need for state and regional planning. JAMA Health Forum. 2020;1(4):e200391. doi:10.1001/jamahealthforum.2020.0391 36218606

